# Supplementary material for: Clinically compliant spatial and temporal imaging of chimeric antigen receptor T-cells
Source: Nat Commun. 2018 Mar 14;9:1081. doi: 10.1038/s41467-018-03524-1 (PMC5852048; doi:10.1038/s41467-018-03524-1)
Supplement: Supplementary file 1 — Supplementary Information [file 41467_2018_3524_MOESM1_ESM.pdf]

Clinically Compliant Spatial and Temporal Imaging of Chimeric Antigen Receptor T-cells

Emami-Shahri et al.

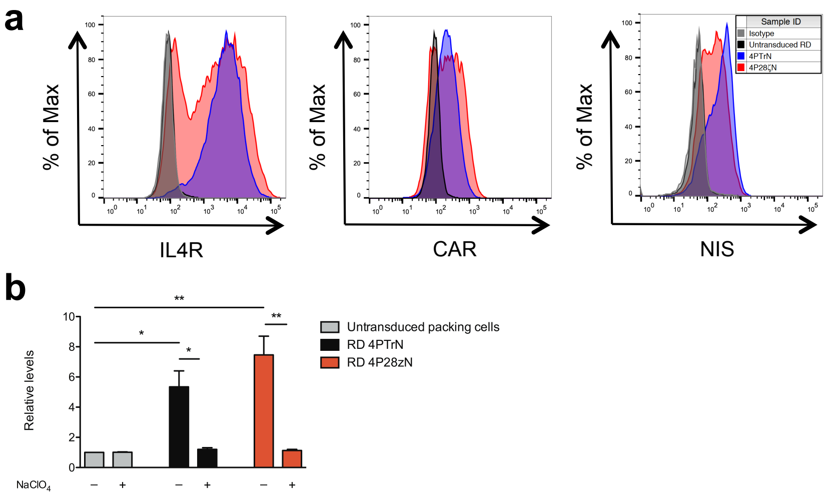

Supplementary Figure 1

Tri-cistronic retroviral vectors validation on stable packaging cell lines.

(a) Representative flow cytometry histograms of 4αβ chimeric cytokine receptor, CAR and hNIS expression on the cell surface of retroviral packaging cell lines. **(b)** Relative *in vitro* uptake levels of 293VEC-RD114 packaging cell lines treated with $^{99m}TcO_4^-$ in the presence or absence of $NaClO_4^-$. Graph presents mean ± s.e.m of three independent experiments. *p<0.05, **p<0.01.

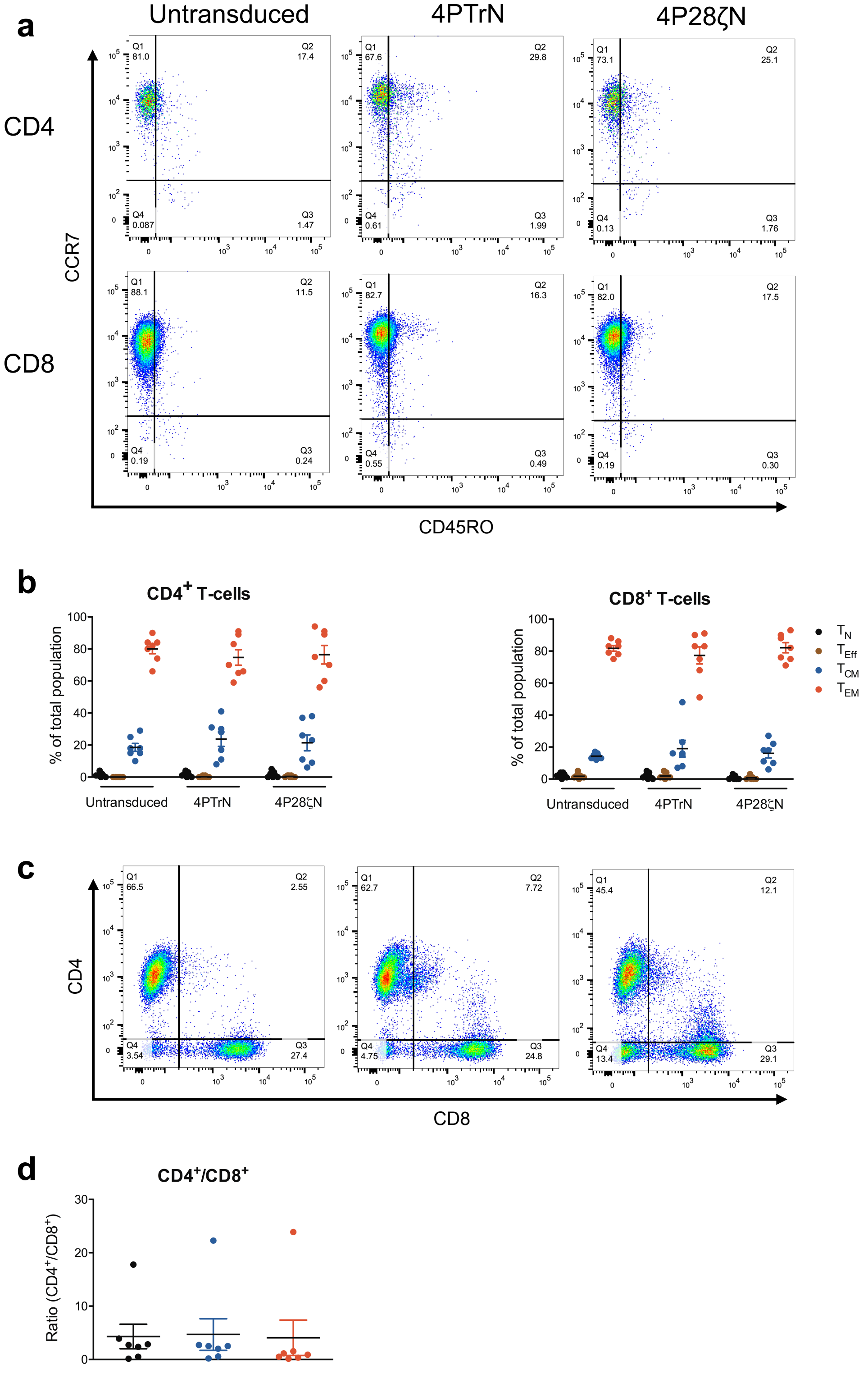

Supplementary Figure 2

Characterization of gene-modified T cells.

Immunophenotyping of T-cells showing naïve ($CD45RO^{+}CCR7^{-}$), effector ($CD45RO^{-}CCR7^{-}$), central memory ($CD45RO^{+}CCR7^{+}$) and effector memory ($CD45RO^{-}CCR7^{+}$) sub-sets by flow cytometry. **(a)** Representative and **(b)** pooled data for CD4 and CD8 subsets for untransduced, $4PTrN^{+}$ and $4P28ζN^{+}$ T-cells. n = 6 donors/group. Graphs present mean + s.e.m. **(c)** Representative and **(d)** pooled $CD4^{+}/CD8^{+}$ ratio data. n = 6 donors/group. Graph presents mean + s.e.m.

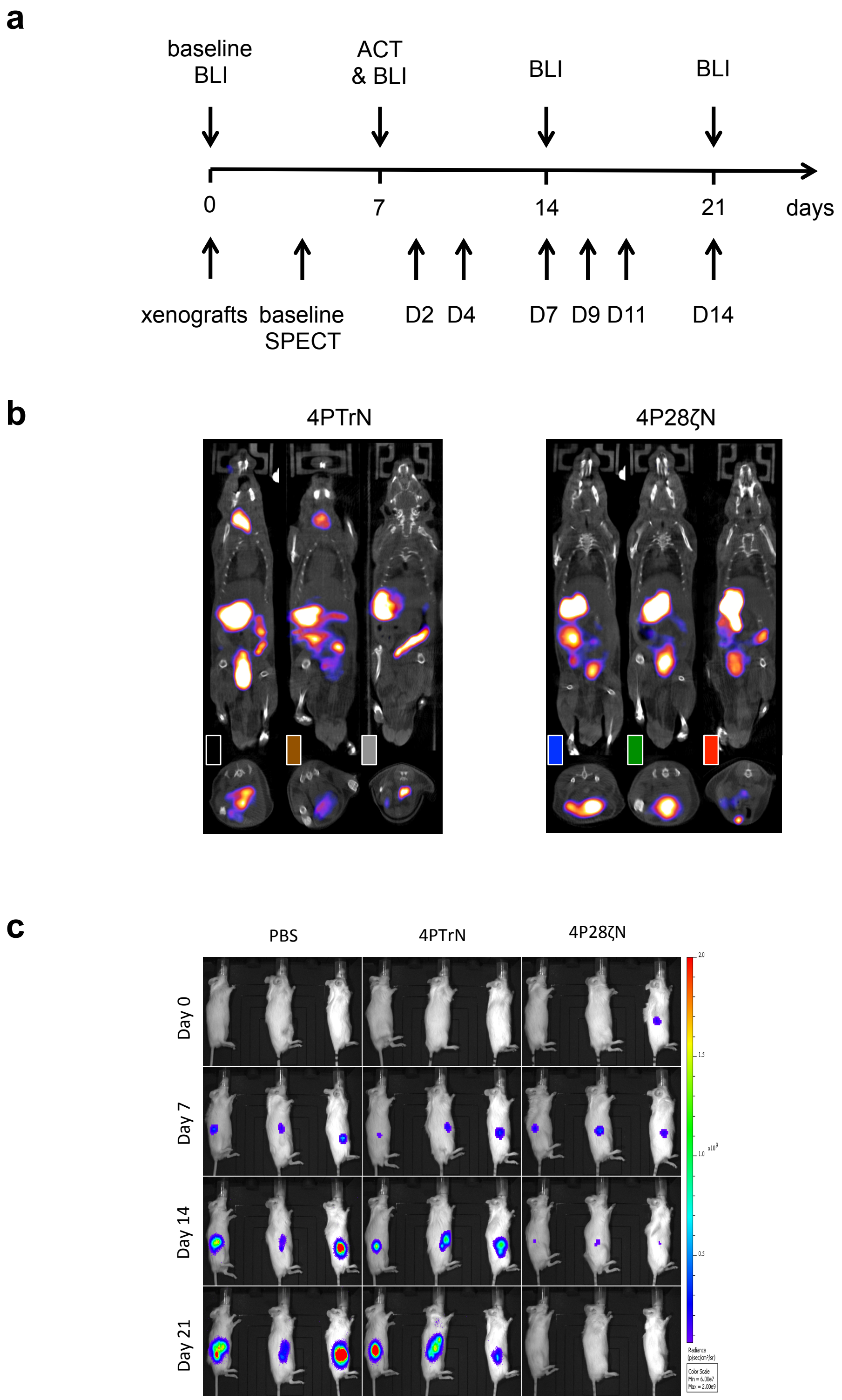

Supplementary Figure 3

Baseline SPECT-CT and BLI images of the 'low tumor burden' model animals.

(**a**) Schematic diagram of the experimental design. (**b**) SPECT-CT images depicting the baseline activity in the individual animals. **(c)** BLI images depicting the tumor progression in all animals.

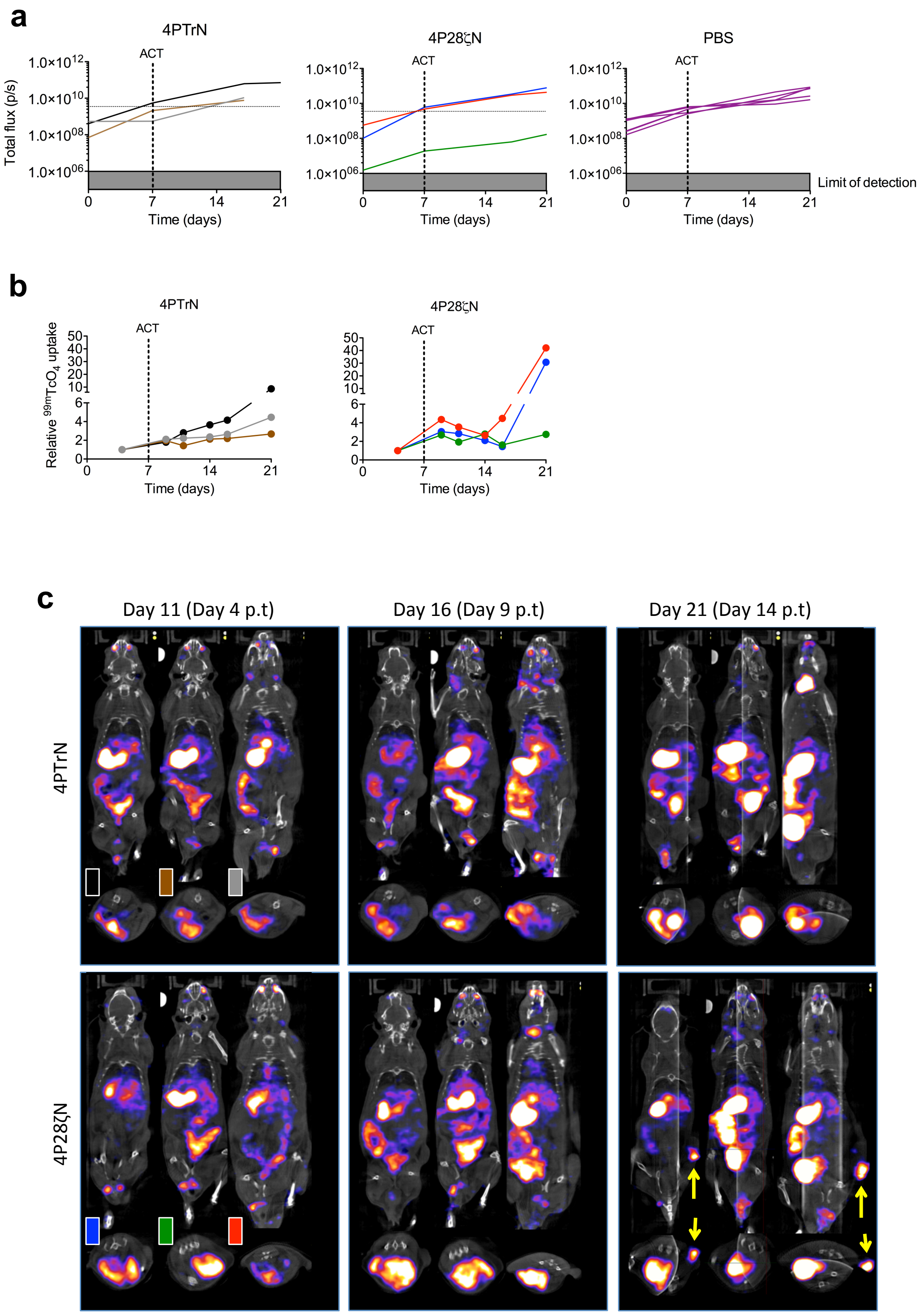
a
4PTrN
4P28ζN
PBS
ACT
Total flux (p/s)
1.0×10^12
1.0×10^10
1.0×10^08
1.0×10^06
Time (days)
Limit of detection
b
Relative 99mTcO4 uptake
c
Day 11 (Day 4 p.t)
Day 16 (Day 9 p.t)
Day 21 (Day 14 p.t)
4PTrN
4P28ζN

Supplementary Figure 4

Further 'low tumor burden' model imaging shows accumulation in tumors through SPECT-CT.

(a) Burden of subcutaneous *firefly luciferase*-expressing PLP tumors was assessed by BLI over a period of 21 days. n = 3 animals/group. Graphs present individual animal BLI signals. **(b)** $^{99m}TcO_4^-$ activity in the tumor was quantified through 3D ROI analysis and normalized to baseline. n = 3 animals/group. Graphs present individual animal SPECT signals. **(c)** SPECT-CT images depicting the accumulation of NIS-expressing T cells in the tumors (indicated by the yellow arrows) of the individual animals (p.t = post-therapy).

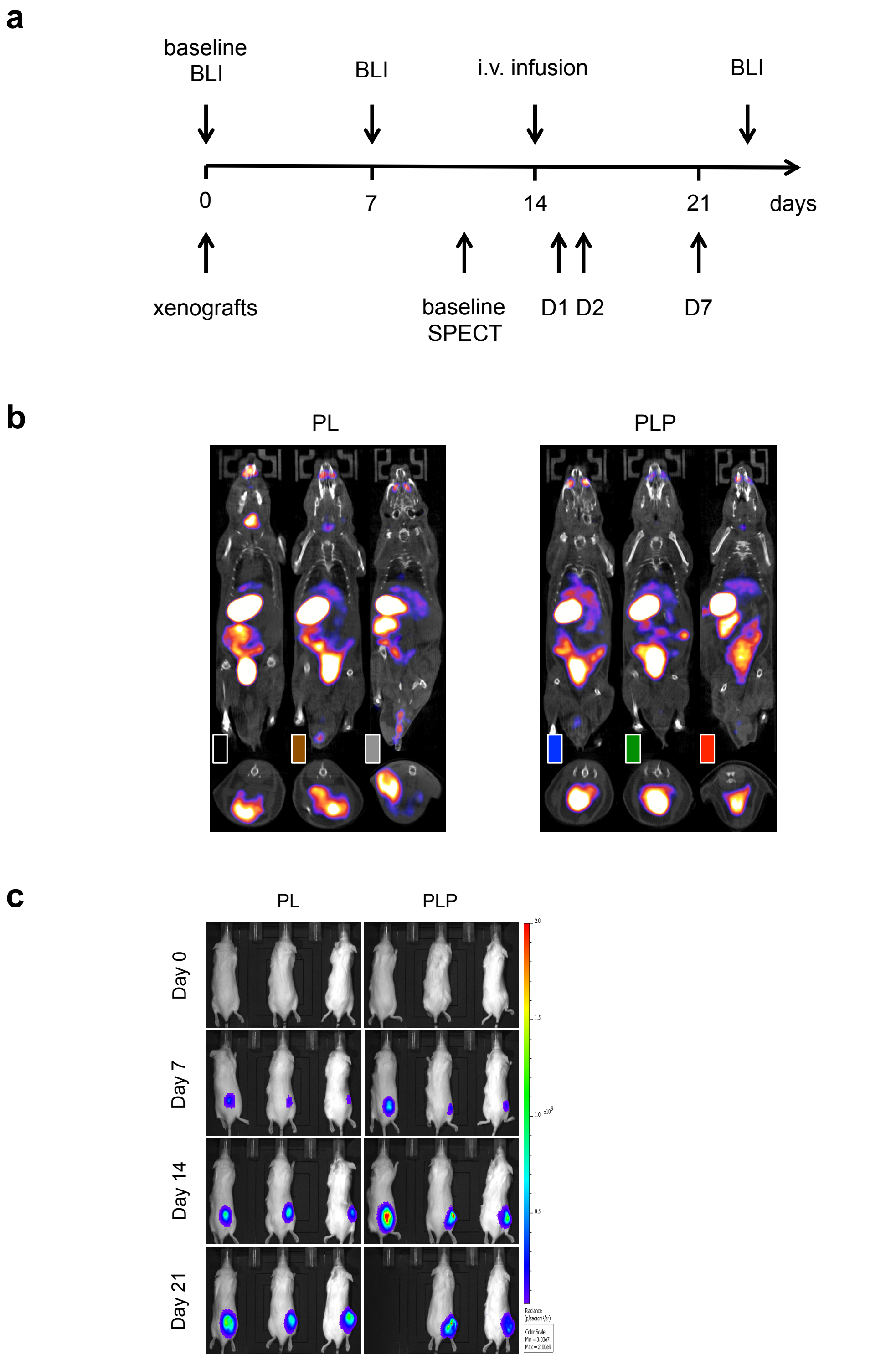

Supplementary Figure 5

Baseline SPECT-CT and BLI images of the 'high tumor burden' model animals.

(**a**) Schematic diagram of the experimental design. (**b**) SPECT-CT images depicting the baseline activity in the individual animals. **(c)** BLI images depicting the tumor progression in treated animals.

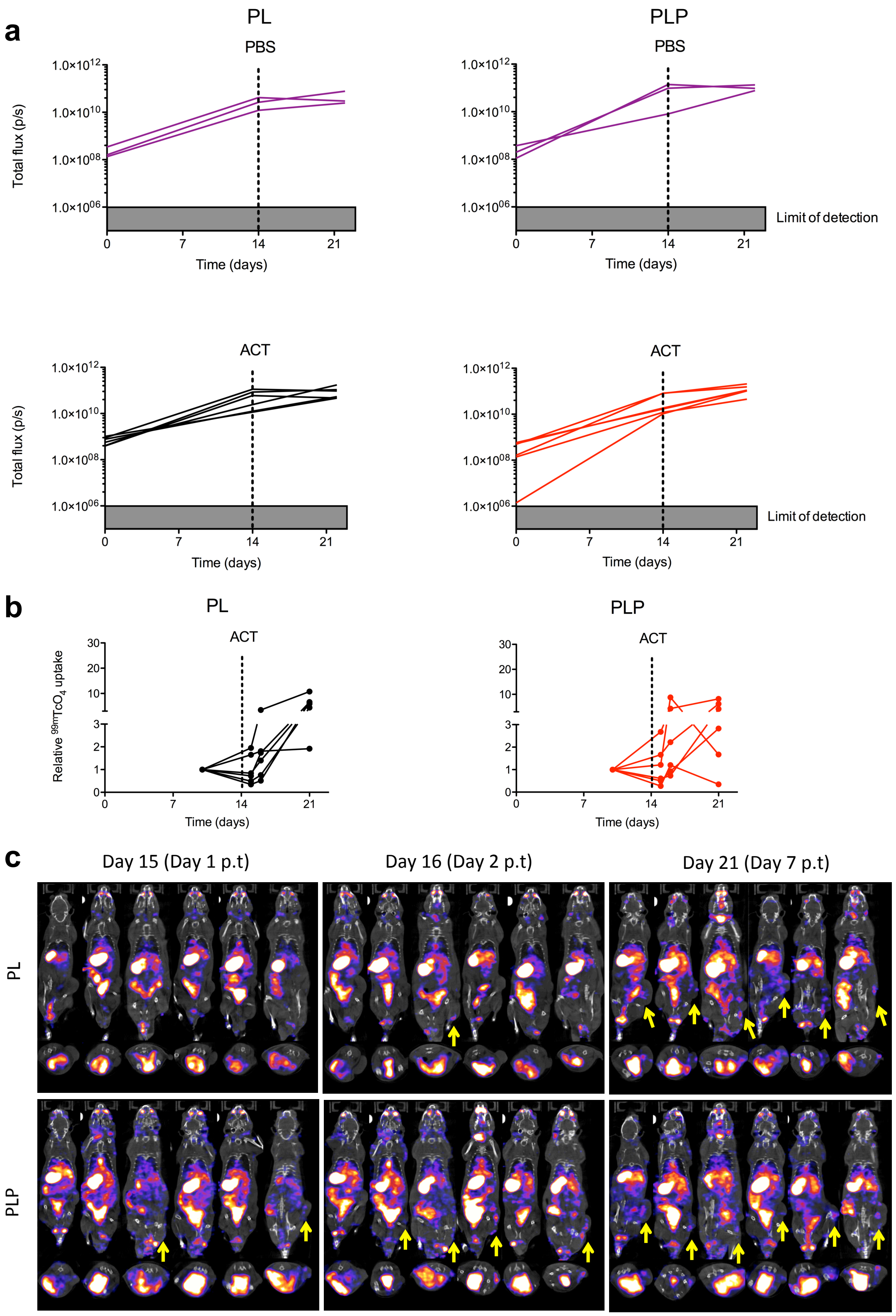

Supplementary Figure 6

Further 'high tumor burden' model imaging shows rapid accumulation of 4P28ζN T-cells within the tumor mass.

(a) Burden of subcutaneous *firefly luciferase*-expressing PL and PLP tumors was assessed by BLI over a period of 21 days. Top panel, PBS treated animals n = 3 animals/group. Bottom panel, 4P28ζN treated animals n = 6 animals/group. Graphs present individual animal BLI signals. **(b)** $^{99m}TcO_4^-$ activity in the tumor was quantified through 3D ROI analysis and normalized to baseline. n = 6 animals/group. Graphs present individual animal SPECT signals. **(c)** SPECT-CT images depicting the accumulation of NIS-expressing T-cells in the individual animals (p.t = post-therapy).

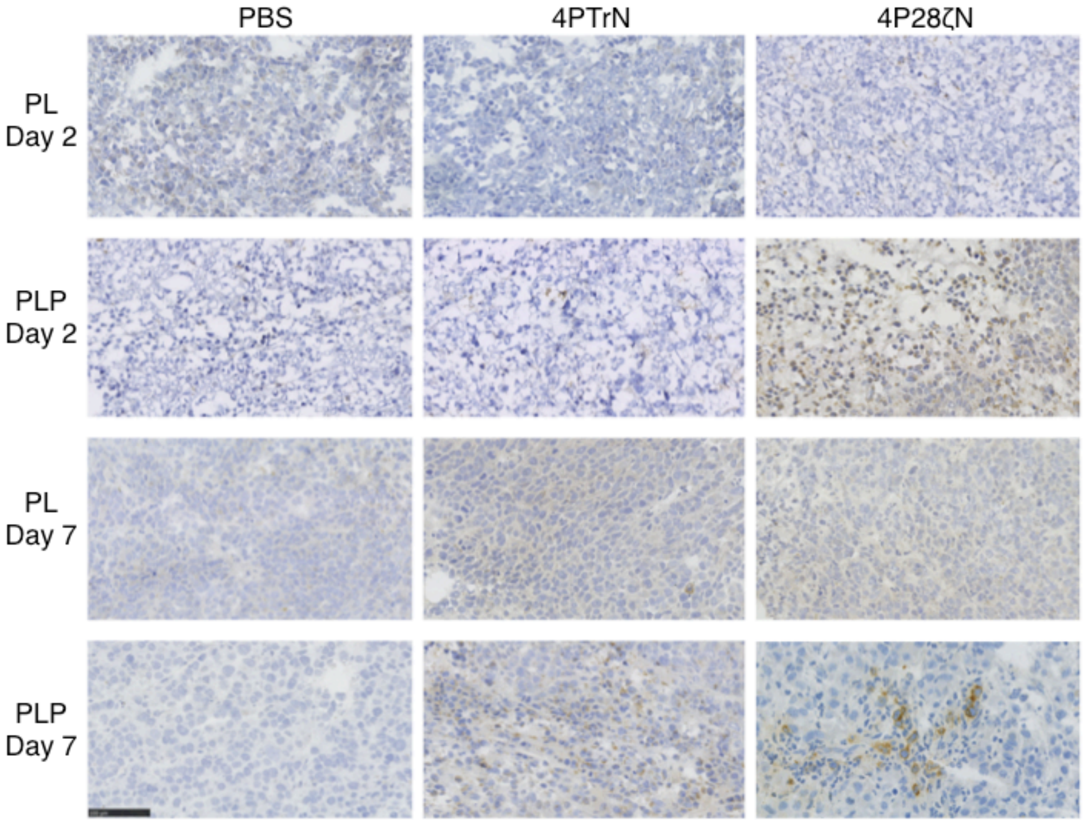

Supplementary Figure 7

CD3ζ staining of T cell infiltrate in 'high tumor burden' model.

Representative IHC images from PL and PLP xenografts harvested and frozen in OCT at 2 or 7 days post i.v injection of PBS, 4PTrN or 4P28ζN T-cells. Sections were stained for human-CD3ζ. n = 3 animals/group.
