# Supplementary material for: Clinically compliant spatial and temporal imaging of chimeric antigen receptor T-cells
Source: Nat Commun. 2018 Mar 14;9:1081. doi: 10.1038/s41467-018-03524-1 (PMC5852048; doi:10.1038/s41467-018-03524-1)
Supplement: Supplementary file 3 — Description of Additional Supplementary Files [file 41467_2018_3524_MOESM3_ESM.pdf]

Description of Additional Supplementary Files

File Name: Supplementary Movie 1
Description: 4PTrN day 4 post T cell treatment.

File Name: Supplementary Movie 2
Description: 4PTrN day 9 post T cell treatment.

File Name: Supplementary Movie 3
Description: 4PTrN day 14 post T cell treatment.

File Name: Supplementary Movie 4
Description: 4P28ζN day 4 post T cell treatment.

File Name: Supplementary Movie 5
Description: 4P28ζN day 9 post T cell treatment

File Name: Supplementary Movie 6
Description: 4P28ζN day 14 post T cell treatment.
