# Supplementary figures and images for: Clinically compliant spatial and temporal imaging of chimeric antigen receptor T-cells
Source: Nat Commun. 2018 Mar 14;9:1081. doi: 10.1038/s41467-018-03524-1 (PMC5852048; doi:10.1038/s41467-018-03524-1)

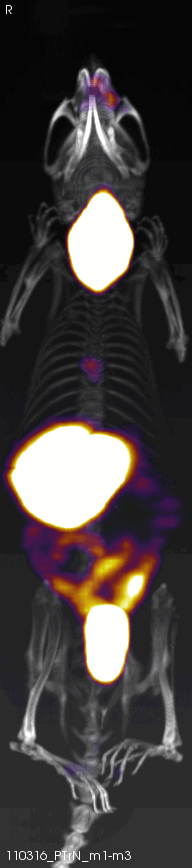

Supplement: Supplementary file 4 — Supplementary Movie 1 [file 41467_2018_3524_MOESM4_ESM.gif]

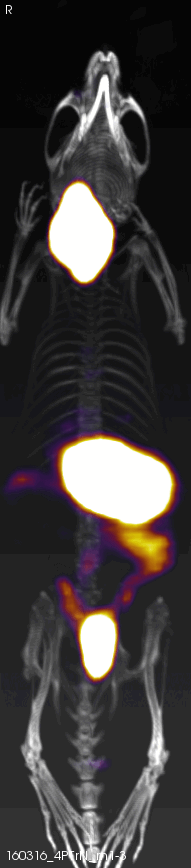

Supplement: Supplementary file 5 — Supplementary Movie 2 [file 41467_2018_3524_MOESM5_ESM.gif]

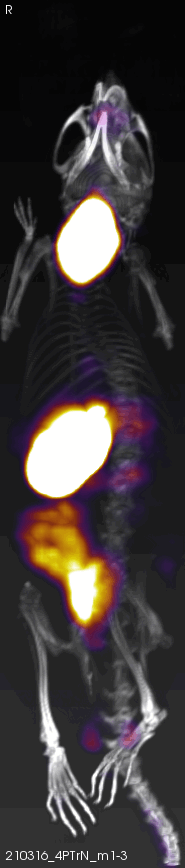

Supplement: Supplementary file 6 — Supplementary Movie 3 [file 41467_2018_3524_MOESM6_ESM.gif]

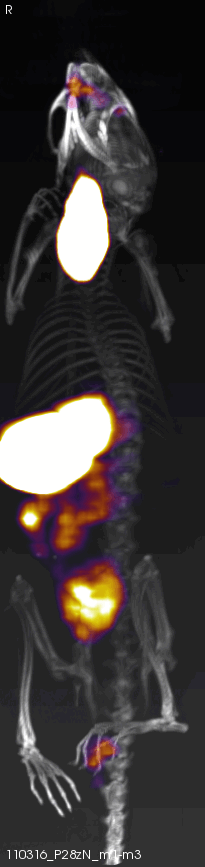

Supplement: Supplementary file 7 — Supplementary Movie 4 [file 41467_2018_3524_MOESM7_ESM.gif]

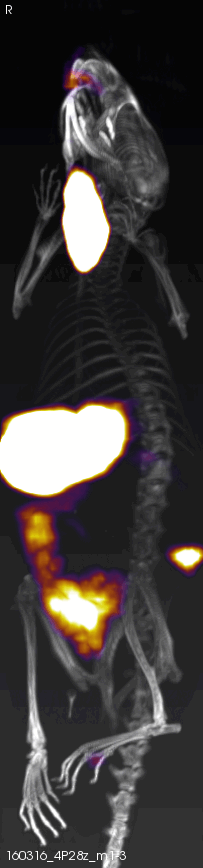

Supplement: Supplementary file 8 — Supplementary Movie 5 [file 41467_2018_3524_MOESM8_ESM.gif]

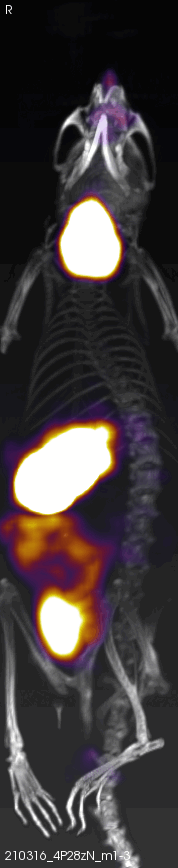

Supplement: Supplementary file 9 — Supplementary Movie 6 [file 41467_2018_3524_MOESM9_ESM.gif]
